# Rapid Evaluation of Antibacterial Carbohydrates on a Microfluidic Chip Integrated with the Impedimetric Neoglycoprotein Biosensor

**DOI:** 10.3390/bios13090887

**Published:** 2023-09-18

**Authors:** Haijie Ji, Xueqiong Yang, Hang Zhou, Feiyun Cui, Qin Zhou

**Affiliations:** The Ministry of Education Key Laboratory of Clinical Diagnostics, School of Laboratory Medicine, Chongqing Medical University, Chongqing 400016, China; jihaijie@stu.cqmu.edu.cn (H.J.); 2021110573@stu.cqmu.edu.cn (X.Y.); 2020111408@stu.cqmu.edu.cn (H.Z.)

**Keywords:** impedimetric biosensor, microfluidic chip, glycoprotein nano-sensing surface, Man-BSA/Au NPs, antiadhesive carbohydrates

## Abstract

The colonization of some bacteria to their host cell is mediated by selective adhesion between adhesin and glycan. The evaluation of antiadhesive carbohydrates in vitro has great significance in discovering new antibacterial drugs. In this paper, a microfluidic chip integrated with impedimetric neoglycoprotein biosensors was developed to evaluate the antibacterial effect of carbohydrates. Mannosylated bovine serum albumin (Man-BSA) was taken as the neoglycoprotein and immobilized on the microelectrode-modified gold nanoparticles (Au NPs) to form a bionic glycoprotein nanosensing surface (Man-BSA/Au NPs). *Salmonella typhimurium* (*S. typhimurium*) was selected as a bacteria model owing to its selective adhesion to the mannose. Electrochemical impedance spectroscopy (EIS) was used to characterize the adhesion capacity of *S. typhimurium* to the Man-BSA/Au NPs and evaluate the antiadhesive efficacy of nine different carbohydrates. It was illustrated that the 4-methoxyphenyl-α-D-pyran mannoside (Phenyl-Man) and mannan peptide (Mannatide) showed excellent antiadhesive efficacy, with IC_50_ values of 0.086 mM and 0.094 mM, respectively. The microfluidic device developed in this study can be tested in multiple channels. Compared with traditional methods for evaluating the antibacterial drug in vitro, it has the advantages of being fast, convenient, and cost-effective.

## 1. Introduction

Studies have shown that the adhesion of pathogenic bacteria to their host cells is frequently mediated by adhesin–glycan interactions [1]. The usage of suitable carbohydrates can inhibit the bacteria adhesion and then the bacterial colonization and biofilm formation [2,3,4]. Hence, carbohydrates have become novel potential drugs for treating bacterial infections. Carbohydrates defend against infectious diseases by preventing bacteria from adhering to host cells without disrupting the microbial community in the gut [5]. They also do not kill the bacteria and affect classic antibiotic targets, which can reduce the bacterial mutation rate and slow the emergence of antibiotic-resistant bacteria [6,7]. Uropathogenic *E. coli* (UPEC) is currently the most studied bacteria–glycan interaction model. Its type 1 pili (containing adhesin FimH) can recognize mannose on the surface of host cells. Lots of chemically synthesized mannoside for UPEC have been reported [1,8]. Their antiadhesive properties were demonstrated by in vivo or in vitro bioassays. *Pseudomonas aeruginosa* is one type of opportunistic pathogen that can cause lung infections. Galactosylated and fucosylated calixarenes were demonstrated to be able to prevent the *Pseudomonas aeruginosa* from adhering to lung cells [9]. 

The hemagglutination inhibition (HAI) assay and the enzyme-linked immunosorbent assay (ELISA) are the most used methods for evaluating the antiadhesive efficacy of carbohydrates in vitro [10]. Although these methods have good reliability, they have the disadvantages of complicated and tedious operation. A surface plasmon resonance (SPR)-based biosensor was also reported to study the binding affinity of FimH and mannosylated dendrimers and evaluate the antiadhesive efficacy [11]. In recent years, microfluidic-chip-based drug evaluation methods have achieved great development owing to their advantages of multiplexing and low reagent usage [12,13]. Microfluidic chips can provide a controllable flow microenvironment that can considerably enhance the binding rate of cells to various biological surfaces [14,15,16]. Meanwhile, microfluidic chips can integrate with all needed functional units in one device and couple with various kinds of detection techniques [17]. Electrochemical impedance spectroscopy (EIS) is a label-free and effective technique to quantitatively detect biological events [18,19,20,21]. The signal changes sensitively with the adsorption and desorption of analytes on the electrode surface. It is a desirable method for detecting binding events of bacteria. Combining microfluidic chips and impedimetric biosensors have a great potential for evaluating the antiadhesive efficiency of carbohydrates. 

Constructing a glycosylated adhesion model on electrodes is critical to developing a desirable impedimetric biosensor on microfluidic chips. Both live human cells [22] and artificial glycosylated surfaces [23] have been reported as adhesion models for the investigation of antiadhesive efficacy in vitro. Compared with human cells, artificially constructed bionic glycosylated surfaces have the merits of being convenient and robust. Hence, the artificial glycosylated surface becomes an ideal glycosylated adhesion model. However, it is noted that designing the artificial glycosylated surface should mimic the presentation of glycans on the host cells. Studies have shown that the binding affinity of a single glycan molecule with bacteria adhesin is generally weak [24,25]. Tuning the density of glycan molecules on the artificial glycosylated surface can facilitate multivalent interactions between glycan and bacteria [26]. Constructing a glycosylated surface on the nanometer scale can better simulate the appearance and arrangement of glycans on the cell surface and also promote the occurrence of multivalent interactions. In addition, on the natural cell surface, glycan generally exists in the form of glycoconjugates (glycoproteins and glycolipids). The adhesin of *S. typhimurium* and *E. coli* (type 1 fimbria, containing adhesin FimH) selectively recognizes glycoproteins on the surface of host cells instead of free glycan molecules [27]. Therefore, a glycoprotein surface can better simulate the glycosylated surface of the host cell. The protein part of the glycoprotein can form a para-binding site with the adhesin [28,29], which generates a higher binding affinity between glycan and bacteria.

In this paper, a microfluidic chip integrated with an impedimetric biosensor is developed for evaluating the antiadhesive efficacy of carbohydrates. A bionic glycoprotein nanosensing surface (Man-BSA/Au NPs) is constructed on the surface of the impedimetric biosensor. According to the selective adhesion of *S. typhimurium* to mannose, the adhesive capacity of *S. typhimurium* to the Man-BSA/Au NPs was detected and analyzed using an impedimetric biosensor with the method of EIS. Furthermore, the antiadhesive efficacy of several different carbohydrates, such as D-Man, Me-Man, Phenyl-Man, and Mannatide is characterized and evaluated. 

## 2. Materials and Methods

### 2.1. Materials and Reagents

D-mannose-BSA (14 atom spacer) was purchased from Dextra Group. 11-mercaptoundecanoic acid (11-MUA, ≥95%), 1-(3-dimethylaminopropyl)-3-ethylcarbodiimide hydrochloride (EDC, AR) and N-hydroxysuccinimide (NHS, AR) were purchased from Aladdin Reagents Co., Ltd. (Shanghai, China). Potassium ferricyanide (K_3_[Fe(CN)_6_], AR), potassium ferrocyanide (K_4_[Fe(CN)_6_], AR), potassium chloride (KCl, AR), and hydrochloroauric acid (HAuCl_4_·4H_2_O, AR) were purchased from Kelong Co., Ltd. (Chengdu, China). LB broth medium, lactose broth medium, and nutritional broth medium were purchased from Land Bridge Technology Co., Ltd. (Beijing, China). Bacteria culture procedures are described in the Supporting Information S1 (SI-1).

Phosphate-buffered saline (PBS, 0.01 M, pH 7.4, including 0.1368 M NaCl, 0.0027 M KCl, 0.0081 M Na_2_HPO_4_, 0.0019 M KH_2_PO_4_) was filtered through 0.22 μm syringe filters (PES) before use. The water was ultrapure water (18.2 MΩ·cm), prepared with a Smart-S super pure water machine (Hitech Instruments Co., Ltd., Chongqing, China).

### 2.2. Preparation of Microfluidic Chip Integrated with Impedimetric Biosensors

**Figure 1:**
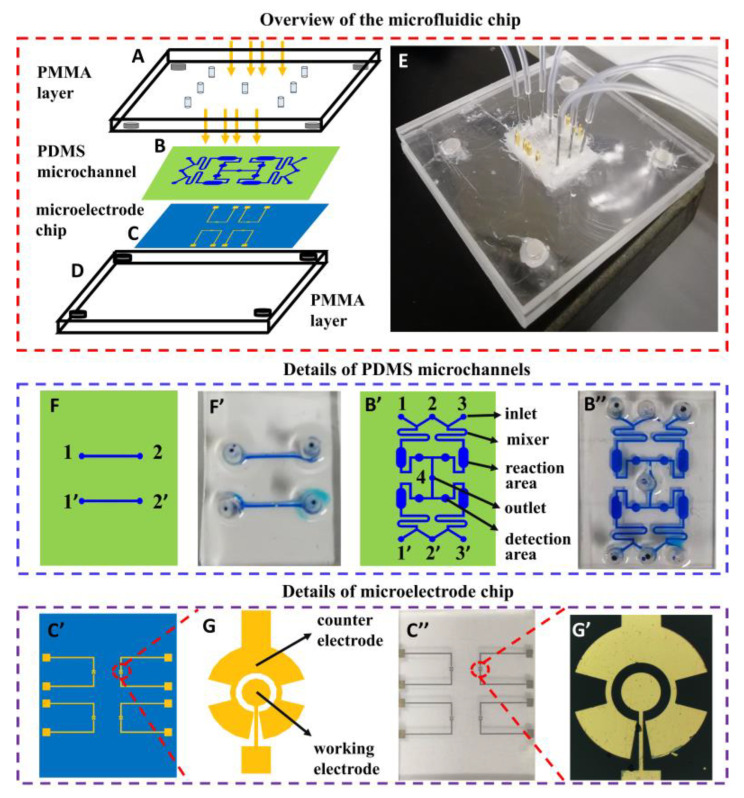
Microfluidic device containing two PMMA layers (**A**,**D**), one PDMS layer with microchannels (**B**) and one glass layer with gold microelectrodes (**C**). (**E**) Image of the whole microfluidic device. (**F**,**F’**) Design drawing and physical image of PDMS with microchannels used for preparing the glycoprotein nanosensing surface on the working electrode while minimizing the modification of the counter electrode. (**B’**,**B”**) Design drawing and physical image of PDMS with microchannels (contain inlet, mixer, reaction area, and detection area) used for testing the antiadhesive efficacy of carbohydrates. (**C’**,**C”**) Design drawing and physical image of the microelectrode chip with 4 pairs of microelectrodes. (**G**,**G’**) Design drawing and physical image of one pair of microelectrodes.

A microfluidic device contains two poly(methyl methacrylate) (PMMA, also known as acrylic) layers (Figure 1A,D), a polydimethylsiloxane (PDMS) layer with microchannels (Figure 1B) and a glass layer with gold microelectrodes (Figure 1C). The Both PMMA layers are used as a holder, which is processed with a laser engraving machine, and the overall size of the microfluidic chip is 20 mm × 25 mm. The microelectrode chips, with 4 pairs of microelectrodes (Figure 1C’,C”) and two molds for replicating PDMS with microchannels, were fabricated using photolithography as described in previous works [30]. The working electrodes are 200 μm in diameter, and the counter electrodes are 350 μm in radius. The working electrode and counter electrode are spaced 50 μm. The width of the connected part is 10 μm (Figure 1G). Figure 1G’ is the image of one pair of microelectrodes. The PDMS with microchannels used for testing the antiadhesive efficacy of carbohydrates is shown in Figure 1B’. Its image is shown in Figure 1B”. The width of the mixing area is 300 μm, the width of the reaction area is 1500 μm, and the radius of the detection area is 500 μm. Another PDMS with microchannels (width in 200 μm), shown in Figure 1F, is used for preparing the glycoprotein nanosensing surface on the working electrode while minimizing the modification of the counter electrode. The image shown in Figure 1F’. Figure 1E is the image of the whole microfluidic device.

### 2.3. Preparation of Man-BSA/Au NPs

Gold nanoparticles (Au NPs) were prepared on the surface of the micro working electrode using the potentiostatic deposition method with a saturated calomel electrode as a reference electrode and a platinum electrode as the counter electrode. The electrolyte solution is a mixed solution of 75 mM KCl and 10 mM HAuCl_4_. The deposition voltage was −0.08 V, and the deposition time was 250 s.

The PDMS with microchannels shown in Figure 1Fwas selected to be reversibly bonded to the microelectrode chip (Figure 1C). A 10 mM ethanol solution of 11-MUA was added from the inlet 1/1′ to modify Au NPs on the working electrode. Self-assembled monolayers (SAMs) of 11-MUA could be formed after 5 min. The carboxyl groups of SAMs were activated with 100 mL of EDC (1%, *W*/*V*) for 10 min at room temperature. In every step, excess agents were thoroughly washed with ultrapure water. The microelectrode containing the activated carboxyl group was immersed in the D-mannose-BSA (14 atom spacer) solution at 1 mg/mL and reacted at room temperature for 2 h. Then, it was immersed in a 3 mg/mL BSA aqueous solution for 30 min to block non-selective binding sites. After all these steps, the glycoprotein nanosensing surface Man-BSA/Au NPs was constructed. 

### 2.4. Adhesive Capacity Test of S. typhimurium to Man-BSA/Au NPs 

The adhesive capacity of *S. typhimurium* to Man-BSA/Au NPs was characterized using EIS with the designed two-electrode system. The PDMS with microchannels shown in Figure 1B’ was bonded to the microelectrode chip with Man-BSA/Au NPs modifying the working electrode. The *S. typhimurium* solution, with a concentration of 1 × 10^4^ CFU/mL, was added from inlet 2/2′ and continued for 20 min at a flow rate of 0.5 uL/min. EIS was measured with the electrochemical system PARSTAT V3 (Princeton Co., Oak Ridge, TN, USA). Then, the mixed solution of 0.1 M KCl and 2 mM K_3_[Fe(CN)_6_]/K_4_[Fe(CN)_6_] (1:1 mixture) was added from the inlet 2/2′ and filled the detection area. EIS, with an alternating potential of 5 mV and frequency range from 1 Hz to 100 kHz, was conducted. 

### 2.5. Antiadhesive Efficacy Test of Different Carbohydrates 

Like in Section 2.4, the PDMS with microchannels shown in Figure 1B’ was used. An *S. typhimurium* solution with a concentration of 1 × 10^4^ CFU/mL was added from inlet 2, 2′. Simultaneously, D-(+)-mannose, α-methyl-D-mannosides, 4-Methoxyphenyl-α-D-mannopyrano-side, and mannan peptides were introduced from inlet 1, 1′, 3, and 3′. Other carbohydrates were added in another microfluidic device. The *S. typhimurium* solution and carbohydrates can be mixed in the mixing area and incubated in the reaction area. The fully mixed solution passed through the detection area at a flow rate of 2 uL/min for 20 min. Then, an electrolyte solution consisting of 0.1 M KCl and 2 mM K_3_[Fe(CN)_6_]/K_4_[Fe(CN)_6_] (1:1 mixture) was introduced at a flow rate of 20 uL/min, and after it filled the detection area, the EIS test was performed.

## 3. Results

### 3.1. Strategy for Evaluating the Antiadhesive Efficacy of Carbohydrates

**Figure 2:**
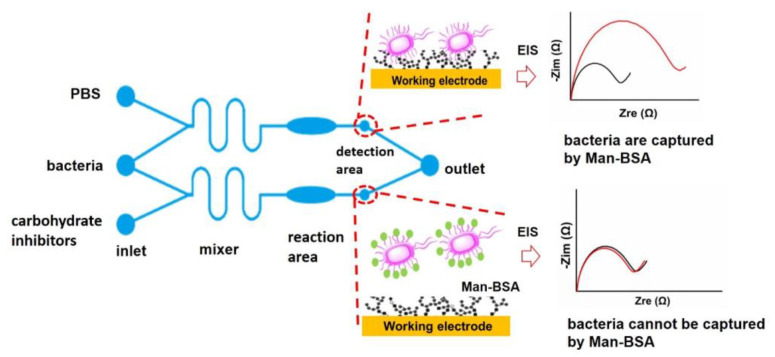
Schematic diagrams of the microfluidic chip containing five functional units, the adhesion of *S. typhimurium* to the Man-BSA/Au NPs, and responses of corresponding EIS Nyquist plots. The black and red lines in Nyquist plots represent EIS before and after adding *S. typhimurium* in the microfluidic chip, respectively.

Developing microfluidic chips for the evaluation of antiadhesive carbohydrates in vitro can facilitate the discovery of new antibacterial drugs. Here, a microfluidic device containing a sample injection area (inlet), mixing area (mixer), reaction area, detection area, and sample output area (outlet) was designed and used to evaluate the efficiencies of adhesive agents for *S. typhimurium* (Figure 2). One pair of microelectrodes with a new structure is integrated into each detection area, and a bionic glycoprotein nanosensing surface (Man-BSA/Au NPs) is constructed on the working electrode while minimizing the modification of the counter electrode. When the *S. typhimurium* solution passes through the detection area, it can selectively adhere to the Man-BSA/Au NPs. The electron transfer resistance (*R*_et_) of the working electrode increases. It appears that the radius of the semicircle increases in the EIS Nyquist plot. When the *S. typhimurium* solution and a solution of an antiadhesive carbohydrate agent are simultaneously added to the microfluidic chip, the carbohydrate agent can bind to the adhesin on the *S. typhimurium* through mixing in the mixing area and incubation in the reaction area. The purpose of incubation was to promote sufficient contact and binding between carbohydrates and bacteria. The carbohydrate-bound *S. typhimurium* cannot adhere to the Man-BSA/Au NPs surface, and the *R*_et_ does not increase (Figure 2). Based on the changes of *R*_et,_ inhibition rates of different carbohydrates can be calculated (Section 3.3).

### 3.2. Optimization of Conditions for Potentiostatic Deposition of Gold Nanoparticles

**Figure 3:**
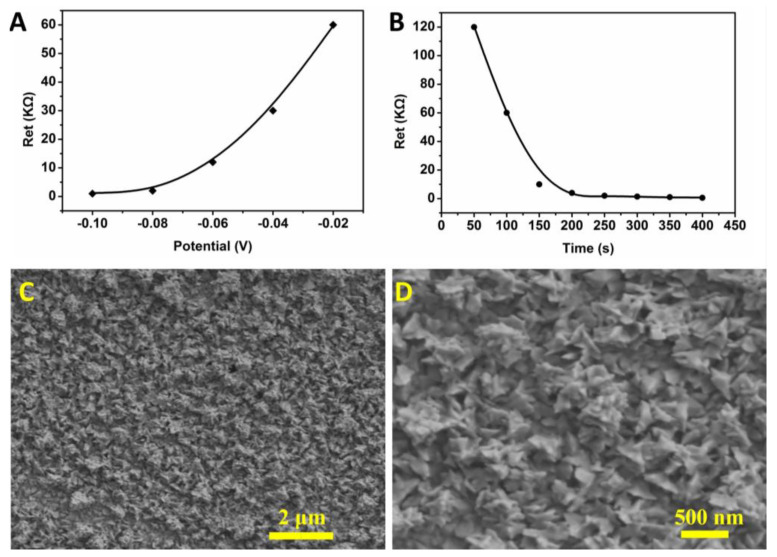
Optimization of conditions for the microelectrode modification of Au NPs; (**A**) optimization of deposition voltage; (**B**) optimization of deposition time; (**C**) SEM image of Au NPs with magnification of 10,000×. (**D**) SEM image of Au NPs with magnification of 30,000×.

The deposition potential and time for the preparation of Au NPs were optimized. The selected conditions for the deposition potential were −0.1 V, −0.08 V, −0.06 V, −0.04 V, and −0.02 V (Figure 3A). The results show that the Au NPs were the most uniform when the deposition voltage was −0.08 V. At this potential, the microelectrode was not damaged. The selected conditions for the deposition time were 50 s, 100 s, 150 s, 200 s, 250 s, 300 s, 350 s, and 400 s (Figure 3B). The results show that the optimized deposition time was 250 s and did not cause a short circuit between the working electrode and the counter electrode. Under optimal conditions, the Au NPs prepared on the surface of the microelectrode had an irregular nano-flower shape, and the particle size of each nano-flower was about 300 nm (Figure 3C,D). Scanning electron microscopy (SEM) images of Au NPs prepared on the microelectrode under voltages of −0.08 V, −0.06 V, −0.04 V, and −0.02 V, with a time of 250 s, are presented in Figure S1. The Au NPs-modified microelectrode can reduce its own electron transfer resistance and increase the specific surface area.

### 3.3. Characterization of Man-BSA/Au NPs and Adhesive Capacity between S. typhimurium and Man-BSA/Au NPs 

**Figure 4:**
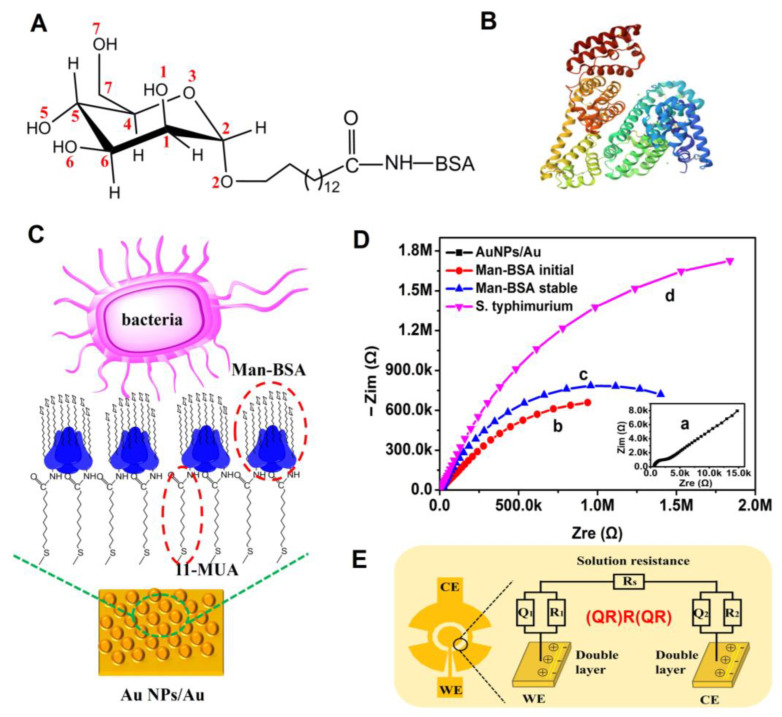
(**A**) Chemical structures of D-mannose-BSA. (**B**) Crystal structure of BSA (PDB: 3V03). (**C**) Schematic diagram of the preparation of the neoglycoprotein nanosensing surface Man-BSA/Au NPs. (**D**) Characterization of the microelectrode and Man-BSA/Au NPs via EIS. (a) Nyquist plot of Au NPs-modified microelectrode, (b) Nyquist plot of Man-BSA fixed on the Au NPs at the initial time, (c) Nyquist plot of Man-BSA/Au NPs incubated in PBS to stable, (d) Nyquist plot after *S. typhimurium* absorbed on the Man-BSA/Au NPs. (**E**) Equivalent circuit model (QR)R(QR) for analyzing the EIS.

A neoglycoprotein nanosensing surface (Man-BSA/Au NPs) was prepared on the gold nanoparticles-modified microelectrode, in which self-assembled 11-mercaptoundecanoic acid was used to conjugate D-mannose-BSA (Figure 4A,B). The D-Mannose part could selectively recognize the adhesin of the *S. typhimurium* [31]. Developing a neoglycoprotein surface on a nano-substrate is beneficial for increasing the surface density of the neoglycoprotein, which can promote the binding efficiency between bacteria and glycan (Figure 4C). Meanwhile, it can increase the active area of the electrode and amplify the impedance signal [32]. The BSA can inhibit the non-selective adsorption of pathogenic bacteria and improve the selectivity. The construction of Man-BSA/Au NPs was characterized using attenuated total reflectance–Fourier-transform infrared spectroscopy (ATR-FTIR). The results are presented in Figure S2. It was confirmed that Man-BSA was successfully immobilized on the Au NPs modified gold electrode using 11-MUA as a linker. An impedance change during the construction of Man-BSA/Au NPs and adsorption of bacteria was recorded and is shown in Figure 4D. An equivalent circuit (QR)R(QR) shown in Figure 4E was used to analyze the EIS. *R_s_* is the resistance of the electrolyte solution. *R*_1_ is the electron transfer resistance of the working electrode. *Q*_1_ is the CPE (Constant Phase Element), which represents the non-ideal capacitance on the working electrode. *R*_2_ is the electron transfer resistance of the counter electrode. *Q*_2_ represents the non-ideal capacitance on the counter electrode. 

### 3.4. Evaluation of Antiadhesive Efficacy of Different Carbohydrates

#### 3.4.1. Qualitative Detection of Antiadhesive Efficacy Using EIS 

The colonization of *S. typhimurium* to their host cell is mediated by selectively recognizing between adhesin and mannose [27,28,33]. Competitively occupying the mannose-binding domain can inhibit the adherence of *S. typhimurium* to epithelial cells. In this study, the neoglycoprotein nano-sensing surface Man-BSA/Au NPs is used as an alternative to the mannose surface of the host cell. The antiadhesive efficacy was evaluated by detecting the inhibition rates of different carbohydrates. Four mannose-containing carbohydrates, i.e., D-mannose (D-Man), methyl α-D-mannopyranoside (Me-Man), 4-methoxyphenyl-α-D-pyran mannoside (Phenyl-Man), and mannan peptide (Mannatide), were tested. Their chemical structural formula is shown in SI-2. D-galactose (D-Gal), L-fucose (L-Fuc), D-glucose (D-Glc), D-xylose (D-Xyl), and sialic acid (D-Sia) were used as negative controls.

An *S. typhimurium* solution at a concentration of 1 × 10^4^ CFU/mL was added from inlets 2 and 2′, and 1 mM solutions of D-Man, Me-Man, Phenyl-Man, Mannatide, D-Gal, L-Fuc, D-Glc, D-Xyl, and D-Sia were added from inlets 1, 1′, 3, and 3′, respectively. *S. typhimurium* and carbohydrates were thoroughly mixed and incubated in the mixing area and the reaction area. An equivalent circuit (QR)R(QR) was used to analyze the EIS, and *R*_et_ could be acquired, and then, the corresponding inhibition rates were calculated using Formula (1).

(1) $$\mathrm{inhibition}\;\left(\%\right)=\frac{R_{\mathrm{et}\left(\mathrm{blank}\right)}-R_{\mathrm{et}\left(\mathrm{mannoside}\;\mathrm{inhibitor}\right)}}{R_{\mathrm{et}\left(\mathrm{blank}\right)}}\times 100\%$$

where the $R_{\mathrm{et}\left(\mathrm{blank}\right)}$ means the *R*_et_ value of just the *S. typhimurium* solution was added to the microfluidic chip. In that case, all the *S. typhimurium* would be attached to the Man-BSA/Au NPs sensing surface, and the *R*_et_ value would be the maximum. $R_{\mathrm{et}\left(\mathrm{mannoside}\;\mathrm{inhibitor}\right)}$ means *R*_et_ values of both *S. typhimurium* solution and various carbohydrate solutions were added into the microfluidic chip. In that case, some mannose-binding sites of *S. typhimurium* would be occupied by the carbohydrate. Reduced *S. typhimurium* could be attached to the Man-BSA/Au NPs sensing surface, and the *R*_et_ value would be decreased. The higher efficiency of the carbohydrate, the lower the *R*_et_ value ($R_{\mathrm{et}\left(\mathrm{mannoside}\;\mathrm{inhibitor}\right)})$. A lower $R_{\mathrm{et}\left(\mathrm{mannoside}\;\mathrm{inhibitor}\right)}$ could induce a higher inhibition (%) based on Equation (1). Hence, Equation (1) could reflect the efficiency of the carbohydrate. The higher the efficiency of the carbohydrate, the higher inhibition (%).

**Figure 5:**
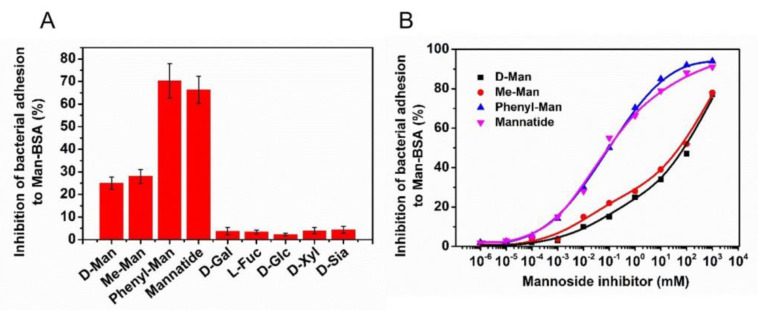
(**A**) Column diagram of the inhibitory rate of different carbohydrates. (**B**) D-Man, Me-Man, Phenyl-Man and Mannatide sigmoidal dose–response curves.

The results show that the inhibition rates of D-Man, Me-Man, Phenyl-Man, Mannatide, D-Gal, L-Fuc, D-Glc, D-Xyl, and D-Sia are 25 ± 2.6%, 28 ± 3.0%, 70 ± 7.6%, 66 ± 6.0%, 3.7 ± 1.7%, 3.4 ± 0.9%, 2.3 ± 0.6%, 3.9 ± 1.4%, and 4.4 ± 1.5%. The histogram is shown in Figure 5A. It is revealed that the mannose agents D-Man, Me-Man, Phenyl-Man, and Mannatide all showed a certain inhibitory effect, while other carbohydrates do not inhibit the adhesion of *S. typhimurium* to the neoglycoprotein nanosensing surface Man-BSA/Au NPs.

#### 3.4.2. Quantitative Evaluation of Antiadhesive Efficacy by IC_50_

**Figure 6:**
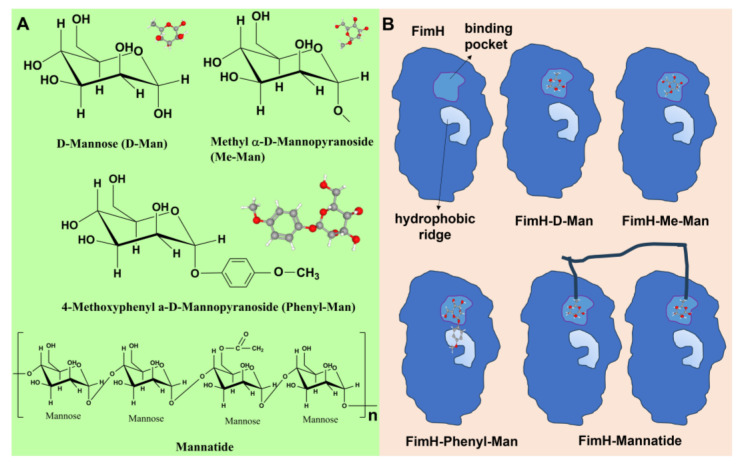
(**A**) Chemical structures and ball–stick structure models of D-Man, Me-Man, Phenyl-Man, and Mannatide. (**B**) Schematic representation of interactions between *S. typhimurium* FimH and Me-Man, Phenyl-Man, and Mannatide, respectively.

In order to quantitatively evaluate the antiadhesive ability of D-Man, Me-Man, Phenyl-Man, and Mannatide, a series of different concentrations of these four mannose agents were prepared and tested. The non-linear fit of the dose–response curve (Figure 5B), using Origin software, was used to determine the IC_50_ (half-maximal inhibitory concentration) of these four mannose containing agents. IC_50_ is the concentration of a particular drug that is needed to inhibit a given biological process to half of the maximum and provides a measure of the effectiveness of the compound. The IC_50_ values of D-Man, Me-Man, Phenyl-Man, and Mannatide were 90 mM, 50 mM, 0.086 mM, and 0.094 Mm, respectively. A lower IC_50_ value indicates a stronger antiadhesive ability. It was shown that the antiadhesive ability of the four mannose agents was Phenyl-Man > Mannatide > Me-Man > D-Man. Phenyl-Man and Mannatide showed excellent antiadhesive efficacy. Mannatide is a kind of mannose polymer. Compared with monosaccharide, it can present higher mannose density near the binding domain of *S. typhimurium*, increase the probability of multivalent interaction, and improve the binding affinity [29,34,35]. Hence, it shows stronger antiadhesive efficacy. The Phenyl-Man molecule has a benzene ring, which can interact with the hydrophobic end around the mannose-binding domain of lectin to form a side binding site [36], which can increase its binding affinity with bacteria. The chemical structures and schematic representation of the interactions are presented in Figure 6. The microfluidic device developed in this study can be tested in multiple channels without culturing cells. Compared with HAI and ELISA, it has the advantages of being fast, sensitive, and simple. 

## 4. Conclusions

A microfluidic chip integrated with an impedimetric neoglycoprotein biosensor for the evaluation of antiadhesive carbohydrates was developed. A neoglycoprotein nanosensing surface (Man-BSA/Au NPs) was constructed on Au NPs-modified microelectrodes. It was demonstrated that *S. typhimurium* can selectively adhere to the Man-BSA/Au NPs using EIS measurement. D-Man, Me-Man, Phenyl-Man, and Mannatide can inhibit the adhesion of *S. typhimurium*. Based on the results of IC_50_, the antiadhesive ability of those four carbohydrates was as follows: Phenyl-Man > Mannatide > Me-Man > D-Man. The microfluidic chip can be applied to other bacteria whose adhesion was mediated by glycan–lectin interactions. For the FimH adhesin-containing bacteria, the microfluidic chip can be employed directly for the evaluation of their antibacterial carbohydrates. For other bacteria, a corresponding specific glycan sensing surface should be developed. The developed microfluidic chip presents a novel platform for evaluating the antibacterial drug in vitro with the merits of being fast, sensitive, and simple. 

## Data Availability

The data presented in this study are available on request from the corresponding author.

## Supplementary Materials

The following supporting information can be downloaded at https://www.mdpi.com/article/10.3390/bios13090887/s1. SI-1: Bacterial Culture; SI-2: SEM images of prepared Au NPs on the microelectrode under different voltages; SI-3: Characterization of nanosensing surface (Man-BSA/AuNPs) using ATR-FTIR; Figure S1: SEM images of prepared Au NPs on the microelectrode under different voltages; Figure S2: Characterization of nano-sensing surface (Man-BSA/AuNPs) by ATR-FTIR. References [37,38,39,40] are cited in the supplementary materials.
