# Supplementary material for: Rapid Evaluation of Antibacterial Carbohydrates on a Microfluidic Chip Integrated with the Impedimetric Neoglycoprotein Biosensor
Source: Biosensors (Basel). 2023 Sep 18;13(9):887. doi: 10.3390/bios13090887 (PMC10526297; doi:10.3390/bios13090887)
Supplement: Supplementary file 1 [file biosensors-13-00887-s001.zip › biosensors-2568888-supplementary.pdf]

Supporting Information

Rapid Evaluation of Antibacterial Carbohydrates on a Microfluidic Chip Integrated with the Impedimetric Neoglycoprotein Biosensor

Haijie Ji, Xueqiong Yang, Hang Zhou, Feiyun Cui and Qin Zhou

SI-1. Bacterial Culture

S. typhimurium ATCC14028 was provided by Professor Yi (Chongqing Medical University). It was cultured in lactose broth at 37 °C for 16 h. The cultured bacteria (1 mL) were collected via centrifugation at 6000 rpm for 5 min. The supernatant was discarded, and the bacteria left at the bottom were dispersed in 1 mL filtered PBS. The centrifugation and washing steps were repeated twice. The cultured cell sample was diluted with filtered PBS to the desired concentrations before use. The number of live cells was determined through colony counting on an agar plate.

SI-2. SEM images of prepared Au NPs on the microelectrode under different voltages

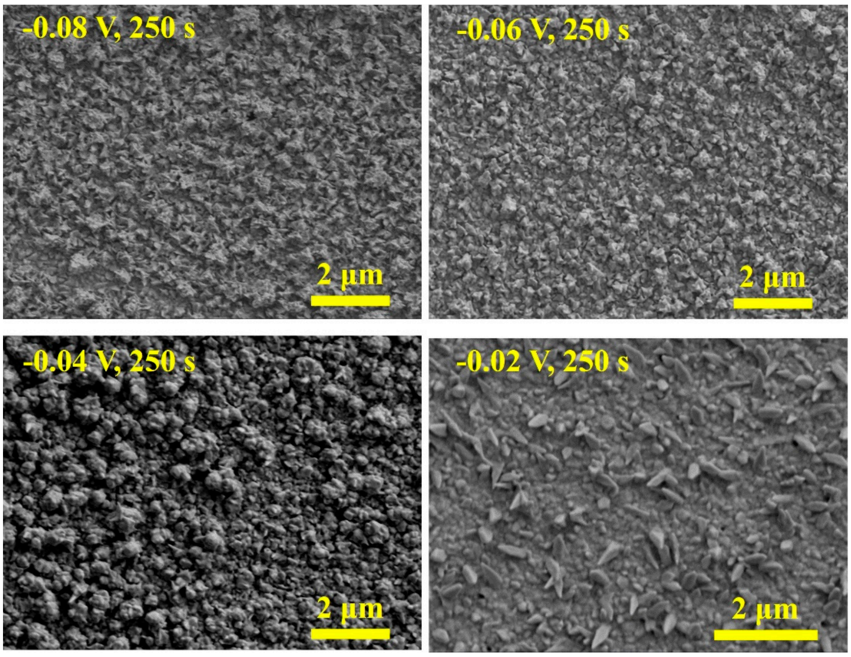

Figure S1. SEM images of Au NPs prepared on the microelectrode under voltages of -0.08 V, -0.06 V, -0.04 V, and -0.02 V, with a time of 250 s.

SI-3. Characterization of nanosensing surface (Man-BSA/AuNPs) using ATR-FTIR

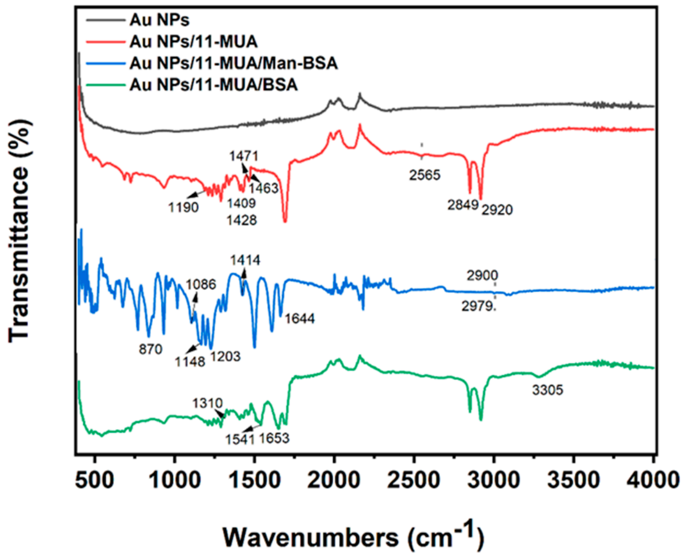

Figure S2. ATR−FTIR spectra of the Au NPs, Au NPs/11−MUA, Au NPs/11−MUA/Man−BSA, and Au NPs/11−MUA/ BSA.

ATR-FTIR of Au NPs/11MUA: The 2920 cm^{-1} peak was assigned to the asymmetric CH_2 stretching. The 2849 cm^{-1} peak was assigned to symmetric CH_2 stretching. The 2565 cm^{-1} peak was assigned to the S-H stretching. The 1409 cm^{-1}, 1428 cm^{-1}, 1463 cm^{-1}, and 1471 cm^{-1} peaks were assigned to the CH_2 scissoring. The 1190 cm^{-1} peak was assigned to symmetric CH_2 stretching [1]

ATR-FTIR of Au NPs/11MUA/BSA: The 1653 cm^{-1} peak was assigned to the amide I band, as expected for a protein with a high proportion of α-helix. The 1541 cm^{-1} peak was assigned to the amide II band [2]. The 1310 cm^{-1} can be assigned to the amide III band [3]

ATR-FTIR of Au NPs/11MUA/Man-BSA: The 2979 cm^{-1} and 2900 cm^{-1} peaks were assigned to mannose C-H stretching vibrations; 1414 cm^{-1} was assigned to mannose C-H angular vibrations; 1644 cm^{-1} was assigned to the stretching vibration of amide bond C=O; 1086 cm^{-1}, 1148 cm^{-1}, and 1203 cm^{-1} were assigned to C-O and C-O-C stretching vibrations on the mannose ring; 870 cm^{-1} was attributed to carbon–nitrogen bond (C-N), which was the characteristic bond in glycoprotein conjugate[4] .

References

1. Elzein T, Fahs A, Brogly M, et al. Adsorption of alkanethiols on gold surfaces: PM-IRRAS study of the influence of terminal functionality on alkyl chain orientation. *J. Adhesion* **2013,** *89,* 416–432. http://dx.doi.org/10.1080/00218464.2013.757521.
2. Retnakumari A, Setua S, Menon D, et al. Molecular-receptor-specific, non-toxic, near-infrared-emitting Au cluster-protein nanoconjugates for targeted cancer imaging. *Nanotechnology*, **2009,** *21,* 055103.
3. Xu Y, Sherwood J, Qin Y, et al. The role of protein characteristics in the formation and fluorescence of Au nanoclusters. *Nanoscale*, 2014, 6, 1515–1524. DOI: 10.1039/c3nr06040c).
4. Wang H, Cheng X, Shi Y, et al. Preparation and structural characterization of poly-mannose synthesized by phosphoric acid catalyzation under microwave irradiation. *Carbohydr.Polym.* **2015,** *121,* 355–361. http://dx.doi.org/10.1016/j.carbpol.2014.12.046.
